# Simulation study of activities of daily living functions using online computerized adaptive testing

**DOI:** 10.1186/s12911-016-0370-8

**Published:** 2016-10-10

**Authors:** Tsair-Wei Chien, Weir-Sen Lin

**Affiliations:** 1 Research Department, Chi-Mei Medical Center, Tainan, Taiwan; 2 Department of Hospital and Health Care Administration, Chia-Nan University of Pharmacy and Science, Tainan, Taiwan; 3 Chi-Mei Medical Center, 901 Chung Hwa Road, Yung Kung Dist, Tainan, 710 Taiwan

## Abstract

**Background:**

Computer adaptive testing (CAT) of the activities of daily living (ADL) functions is required (i) to reveal the advantages of using an efficient and accurate estimation method, (ii) to determine the cutpoint for classifying ADL strata in patients with stroke, and (iii) to evaluate the feasibility of online CAT used in clinical settings for smartphones.

**Methods:**

Normally standardized distributions of ADL measurements were simulated using item parameters from published papers. We retrieved item parameters of the combined Barthel Index and Frenchay Activities Index from the literature (the 23-item comprehensive ADL [CADL] and 34-item ADL scales) and simulated three 1000-person measures from a normal standard CAT distribution: [i] CADL (CADL-CAT), [ii] ADL (ADL-CAT), and [iii] NAT (Non-Adaptive Testing). The cutpoints of ADL person strata were determined using a norm-reference method. *Maximum a posteriori* estimation, *expected a posteriori* estimation, and *maximum likelihood estimation* (MAP) were used to compare the Pearson correlation coefficients and different number ratios of paired measures yielded by CAT and NAT. The number of items and the cutpoints for the scale were separately determined.

**Results:**

We found that (i) correlation coefficients for the three CAT-estimated measures were 0.77 (CADL), 0.93 (Male ADL), and 0.93 (Female ADL) compared with their NAT counterparts. Different number ratios of person-paired measures between CAT and NAT for the three scales were all less than 5 %, indicating no difference exists between CAT and NAT. However, CAT might be 66 % more efficient than NAT. (ii) The estimated cutpoints of T scores (i.e., with a mean of 50 and a standard deviation of 10) were 45, 55, and 65 (e.g., separating person ADL function to four strata with *not active*, *fairly active*, *active*, and *very active*). (iii) An available-for-download online ADL-CAT APP for clinical practice was demonstrated.

**Conclusions:**

An online ADL-CAT APP using the MAP method was created and used on smartphones to classify ADL strata in patients with stroke.

**Electronic supplementary material:**

The online version of this article (doi:10.1186/s12911-016-0370-8) contains supplementary material, which is available to authorized users.

## Background

Physical therapists (PTs) help patients overcome functional disabilities in their physical and social environments [1, 2]. They use a variety of functional scales to evaluate the functional levels of the instrumental activities of daily living (IADL) of their patients. The psychometric properties of these scales have been validated for use in patients with stroke [3–7]. However, most of them merely report numerical results that are not translated into the degree (or classification) of their functional problems relative to a cutpoint (e.g., separating person ADL function strata with *not active*, *fairly active*, *active*, and *very active*) that can be generalized to other healthcare sites and other samples.

### Cutpoints must be determined for patients

Specifically, activities of daily living (ADL) function assessment consist of two scales: (i) the Barthel Index [BI]) [3] and (ii) the Frenchay Activities Index [FAI]) [8]. Because the former tends to have ceiling effects [9], and the latter to have floor effects [7, 10], several authors [7, 11, 12] have recommended combining the two to assess the broad spectrum of ADL functions. Although such a combined scale theoretically overcomes the drawback of floor and ceiling effects [5, 7, 13, 14], none reports cutpoints for the scale. Cutpoints can be used for PTs and patients to identify the degree of patient functional problems.

### Patients are required to answer fewer questions without compromising precision

Combining the two indices seems inefficient because together they require more than 20 min to complete [5, 15, 16]. This means that the traditional non-adaptive testing (NAT) questionnaires have a large respondent burden because they require patients to answer questions that provide no additional information for person estimation [17]. Thus, some researchers [2] recommend using computer adaptive testing (CAT) scales to reduce the patient burden. As with all forms of Web-based technology, advances in mobile health (mHealth) and health communication technology are rapidly increasing [17]. So far, however, there is no online ADL CAT assessment for smartphones.

### Computer programmers must choose suitable types of CAT estimation methods

Item response theory (IRT)-based CAT has been proposed [2, 18–21] for efficient, reliable, and valid assessments of health-related functions. Although many researchers have contributed to the dichotomous [2, 7], polytomous [22, 23], and combined item-bank formats used by CAT (called a Rasch partial credit model [PCM] [24] or a generalized partial credit model [GPCM] [25]), few were jointly available for a comparison of precision and efficiency differences in CAT estimation methods (e.g., *maximum likelihood estimation* [MLE] [26], expected *a posteriori estimation* [EAP] [27, 28], and *maximum a posteriori estimation* [MAP] [29]).

### Study aims

The aims of the current study were to (i) compare CAT and NAT precision and efficiency, (ii) determine the cutpoints of ADL person strata, and (iii) design an online ADL-CAT assessment APP for smartphones.

## Methods

### Study data yielded from simulation data of three kinds of response patterns

The item parameters were retrieved from both the combined 23-item comprehensive ADL/(CADL) [7] and the 34-item ADL for males and females [5]. We assumed that all patients’ true scores follow a normal distribution according to reference [5] (cf. http://ptjournal.apta.org/content/93/5/681/F1.large.jpg). When 1,000 persons’ true scores (sampled from a normal distribution [~*N*(0,1)]) and item difficulties (retrieved from previously published articles [5, 7]) were known (Tables 1 and 2), we simulated three kinds of response pattern datasets using the IRT probability modeling method [30, 31]. A CADL (1000 persons × 23 items) and an ADL (1000 persons × 34 items) for males and females, respectively, were then generated (see spread sheets: main and simulation in Additional file 1).

**Table 1:** Item bank used for ADL-CAT

GPCM parameters for discrimination (D) and threshold step difficulties				
Item bank for ADL-CAT	D (M/F)	Step 1 (M/F)	Step 2	Step 3
1. Washing face	2.18	−1.36		
2. Brushing teeth	1.73	−1.57		
3. Climbing stairs (up and down 1 story)	1.14	−0.47		
4. Walking outside (in the neighborhood for > 15 min)	0.8	−0.84		
5. Taking public transportation	0.52	−0.11		
6. Preparing light meals	1.28/2.6	0.68/0.3		
7. Preparing ingredients for meals	2.71/1.9	1.36/0.81		
8. Washing dishes	2.07/3.65	1.19/0.73		
9. Trash disposal	1.28/2.16	1.38/0.77		
10. Taking out the trash	1.45/4.31	1.5/1.07		
11. Washing clothes	2.12/1.93	1.23/0.65		
12. Using a telephone	0.83	−0.78		
13. Social outings	0.56	0.53		
14. Reading newspapers	0.56	0.48		
15. Reading books	0.57	1.13		
16. Using a computer	0.7	1.64		
17. Art activities	0.78	3.3		
18. Playing board games/cards	0.62	2.62		
19. Singing karaoke	0.63	2.24		
20. Going to a spa (mainly for hot/cold water treatments)	0.96	2.64		
21. Withdrawing money	1.18	1.1		
22. Volunteer work	0.66	3.54		
23. Gainful work	0.73	2.53		
24. Drinking	0.82	−2.17	−1.12	
25. Eating	1.06	−1.95	−0.78	
26. Bladder management	3.23	−1.01	−0.36	
27. Bowel management	3.02	−1.12	−0.24	
28. Getting up	1.95	−1.6	−0.59	
29. Walking inside the house	1.77	−1.31	−0.36	
30. Taking medicine on time	0.93	−1.73	−0.09	
31. Watching television	0.55	−2.48	−1.35	
32. Putting on clothes	2.07	−0.83	−0.03	0.16
33. Taking off clothes	2.36	−0.87	−0.15	0.04
34. Putting on trousers/skirts	2.51	−0.77	−0.12	0.07

*ADL-CAT* activities of daily living-Computerized Adaptive Testing, *GPCM* generalized partial credit model

**Table 2:** Item bank used for CADL-CAT

Rasch model item difficulties (delta)	
Item bank for CADL-CAT	delta
FAI 13: household/car maintenance	4.73
FAI 14: reading books	4.72
FAI 15: gainful work	4.01
FAI 12: gardening	3.75
FAI 9: actively pursuing hobbies	3.53
FAI 11: travel outings/car rides	3.52
FAI 1: preparing main meals	3.24
FAI 3: washing clothes	3.19
FAI 2: washing up	3.09
FAI 5: heavy housework	2.75
FAI 4: light housework	1.95
FAI 10: driving a car/bus travel	1.83
FAI 6: local shopping	0.59
BI 2: bathing	0.55
BI 10: climbing stairs	−0.72
BI 4: dressing	−0.77
BI 9: mobility	−2.85
BI 7: toileting	−3.48
BI 8: transfer	−3.99
BI 3: grooming	−6.77
BI 6: bladder control	−7.09
BI 5: bowel control	−7.33
BI 1: eating	−8.41

*CADL* comprehensive activities of daily living-Computerized Adaptive Testing

### Tasks to reach the Aims

#### Three types of CAT estimations to compare CAT precision and efficiency

Three algorithms—MLE, MAP, and EAP—are commonly used to estimate person measures within the CAT framework. The predominant method is called MLE because it simply finds the highest point on the likelihood function and returns the value at which it occurs. A common variant of this is the Bayesian model estimation procedure, also called MAP, where this likelihood function is multiplied by an additional curve that represents an assumed population distribution. A further variant is to take this Bayesian-modified curve and find, rather than the maximum point, the average value as weighted by the function. This is referred to as Bayesian expectation *a posteriori* (EAP) estimation. We used these three estimation methods to compare CAT with NAT on precision and efficiency. We ran an author-made VBA (Visual Basic for Applications) module in Microsoft Excel to conduct the simulation study (see spread sheets: MLE and EAP in Additional file 1 and eap in Additional file 2).

We used CAT stop rules, e.g., when person reliability reaches 0.90 (= [1 − SEM_pi_] [3], where SEM_pi_ = person standard error of measurement on item i = 1/variance_pi_ = 1/information_pi_), and the last three average consecutive person estimation change is < 0.05 after the minimal necessarily completed number of items is ≥ 7, as proposed in the study [5].

The MLE, MAP, and EAP CAT algorithms were used to (i) estimate person measures on the three kinds of response datasets, (ii) compute correlation coefficients between estimated person measures (a. CADL, CADL_CAT, b. Male ADL, Male ADL_CAT, c. Female ADL, and Female ADL_CAT), (iii) analyze the CAT efficiency of item length shortened by CAT compared with NAT, and, using independent *t* tests to count differences in ratios < 5 % (iv) test whether the precision was equal to NAT [32].

#### The cutpoints of ADL person strata determined using a norm-referred method

**Fig. 1:**
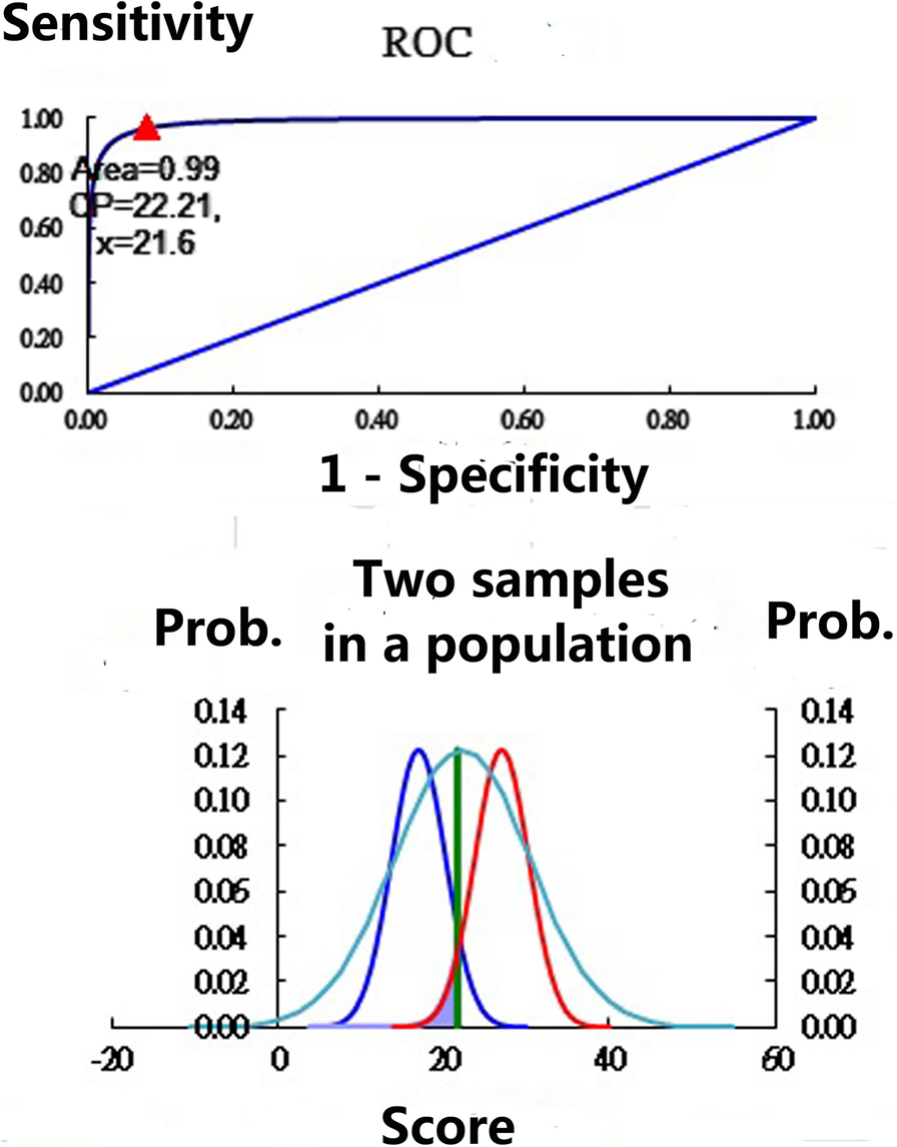
The cutpoints of person strata determined using a norm referred method

Traditionally in clinical practice, researchers use ROC (receiver operating characteristic) curves to plot the true-positive rate (sensitivity) against the false-positive rate (1 − specificity) at various threshold settings [33] (e.g., Fig. 1 with two samples). The preliminary condition is to know the patient’s classification (i.e., stratum) (e.g., separating person ADL function strata with *not active*, *fairly active*, *active*, and *very active*) before conducting the ROC of any two adjacent samples. Unfortunately, we usually do not know the patient’s true- and false-positive disease-specific status. How to determine the so-called gold standard test (e.g.,a cutting point) is an important issue we face in clinical settings in a bit to identify the degree of patient functional problems.

Fortunately, according to the literature [34–36], as a scale’s reliability (i.e., Cronbach’s α) increases, so does the person-number of ranges that can be confidently distinguished. Person measures with a reliability of 0.67 will tend to classify two groups with 95 % confidence; 0.80 will group three groups; 0.90 will separate four groups; 0.94, within five groups; 0.96, within six groups; 0.97, within seven groups; and so on [37]. Thus, the number of person strata for both CADL and ADL can be divided into four groups because each scale’s reliability coefficient is greater than 0.90 [5], which is similar to the Turnbull et al. [8] definition of the four strata (Not Active, Fairly Active, Active, and Very Active) for the Frenchay Activities Index.

We picked up any two adjacent normal distribution samples using the Microsoft Excel function = NORMDIST (*mean*, standard deviation [SD], TRUE), whereas the *mean* is the cluster center obtained using the k-mean method when the number of strata is known according to the Cronbach’s α scale [34], and the SD is obtained from the individual scores of the specific cluster. Using a brute force search of the two adjacent samples, the cutpoint can be determined at the maximal summation of specificity and sensitivity across all possible scores. Interested readers can refer to the Excel spreadsheet *homepage_B* in Additional file 1 for detailed information.

#### An online ADL-CAT assessment APP was designed for use on smart phones

An online routine was designed for patients to report their ADL T scores (mean = 50, SD = 10) that were transformed using the formula: (50 + 10 × estimated person measure for cut-points). The three kinds of item pool (Tables 1 and 2) were uploaded to the website. The first CAT item will be randomly selected from the item pool. The next item to be answered is the item with the maximal variance among the remaining items according to the provisional person ability [21, 38]. For the detailed item selection rules, interested readers can see Additional file 3 on the Excel VBA codes. All the responses will be automatically saved on the study website (see the spreadsheet reply in Additional file 4).

### Statistical tools and data analyses

SPSS 15.0 for Windows (SPSS Inc., Chicago, IL) and MedCalc 9.5.0.0 for Windows (MedCalc Software, Mariakerke, Belgium) were used to calculate (1) Cronbach’s α, (2) dimension coefficients (DCs) [39], and (3) residual DCs [39] on the three kinds of response datasets as well as (4) correlation coefficients between estimated person measures for CAT and NAT. Independent *t* tests were used to compare (5) the ratios of the different paired person measures and to determine (6) cutpoints at maximal summations of specificity and sensitivity for each person stratum when strata central points were determined using k-mean cluster analysis.

## Results

### Task 1: CAT precision and efficiency compared using three estimation methods

The three coefficients (i.e., Cronbach’s α [DC, residual DC]) were 0.61[0.67, 0.49] for the 23-item CADL, 0.90 [0.80, 0.50] for the Male ADL, and 0.90 [0.74, 0.48] for the Female ADL (Table 3), which indicated that these three simulated datasets were unidimensional (i.e., DC ≥ 0.67 and residual DC ≤ 0.56) [39].

**Table 3:** Correlation coefficients (left lower triangle) and different number ratios (right upper triangle) between scales’ estimated measures

Estimation methods	A	B	C	D	E	F
MAP						
A. CADL	0.61	0.40 %				
B. CADL_CAT	***0.77***					
C. Male ADL	**0.67**	0.68	0.90	0.00 %		
D. Male ADL_CAT	0.64	0.64	***0.93***			
E. Female ADL	**0.67**	0.68	1.00	0.93	0.90	0.00 %
F. Female ADL_CAT	0.64	0.64	0.93	1.00	***0.93***	
EAP						
A. CADL	0.67	0.10 %				
B. CADL_CAT	***0.76***					
C. Male ADL	**0.78**	0.91	0.80	0.00 %		
D. Male ADL_CAT	0.75	0.87	***0.95***			
E. Female ADL	**0.78**	0.97	0.94	0.90	0.74	0.00 %
F. Female ADL_CAT	**0.76**	1.00	0.91	0.87	***0.97***	
MLE						
A. CADL	0.49	0.00 %				
B. CADL_CAT	***0.76***	0.00				
C. Male ADL	**0.77**	0.91	0.50	0.00 %		
D. Male ADL_CAT	0.75	0.87	***0.95***			
E. Female ADL	**0.77**	0.97	0.94	0.90	0.48	1.20 %
F. Female ADL_CAT	0.76	1.00	0.91	0.87	***0.97***	

*ADL-CAT* activities of daily living-Computerized Adaptive Testing, *CADL* comprehensive activities of daily living-Computerized Adaptive Testing, *MLE* maximum likelihood estimation, *EAP* expected *a posteriori* estimation, *MAP* maximum *a posteriori* estimation

Scale reliability coefficients shown on the diagonal line from left to right in the upper MAP table; dimension coefficients displayed in the middle EAP table; model’s residual dimension coefficients displayed in the bottom MLE table

Bold-italic values are correlation coefficients (CC) between NAT and CAT. Bold ones show the binary NAT CADL has significant lower CCs (<0.80) than those CCs (>0.90) between polytomous scales

The correlation coefficient between person-estimated measures of CAT and NAT using the MAP method was 0.77 for the CADL, 0.93 for the Male ADL, and 0.93 for the Female ADL, not significantly different from the 0.76, 0.95, and 0.97, respectively, using EAP and MLE) (Table 3).

**Fig. 2:**
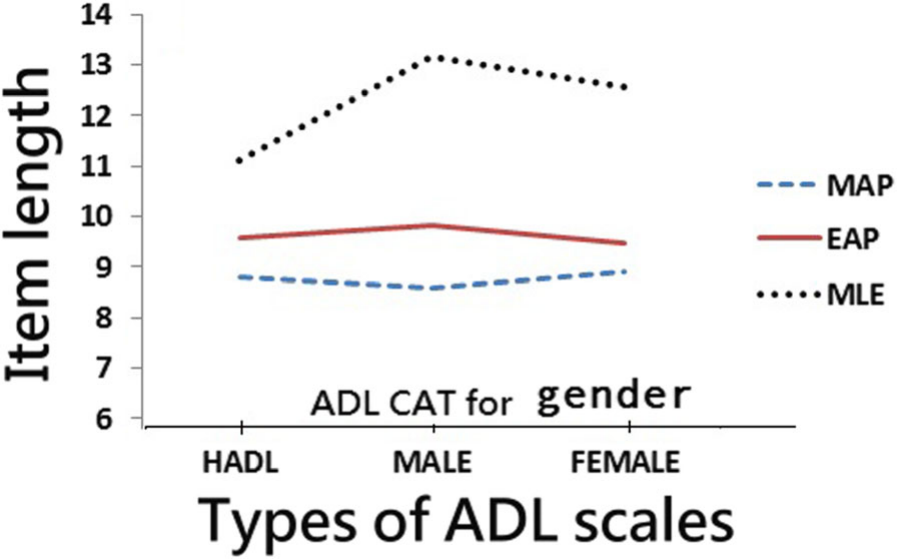
Item length consumed by CAT estimation methods on study scales

The differences in the number ratios between the measures of CAT and NAT using the three estimation methods were all less than 5 %. The item lengths were shorter (Fig. 2). Using CAT, almost 62 % (= [34–13] = 21/34) of the item lengths were shortened. The largest number of items consumed by CAT was when using the MLE method, because it is relatively unbiased and has a well-designed item pool, but it also has a large standard error (SE) relative to the Bayesian MAP and MLE methods [26].

### Task 2: Cutpoints of CADL and ADL

Cutpoints for CADL were 42, 56, and 69; and for ADL were 43, 55, and 65 for males, and 43, 55, and 67 for females. For simplicity, the T scores of cutpoints suggested were at 45, 55, and 65. A four-person stratification (e.g., separating person ADL function strata with *not active*, *fairly active*, *active*, and *very active*) can be well separated (Table 4). All values of specificity and sensitivity were greater than 0.90 (Table 4).

**Table 4:** Determination of cutpoints for the ADL scales

Estimation methods & scales			Strata	Cutpoints			
Characteristics			*n*	IRT-score	*T*-score	Specificity	Sensitivity
MAP	Male ADL	Not Active	285				
		Fairly Active	385	−0.66	43.40	0.96	0.96
		Active	249	0.46	54.60	0.93	0.94
		Very Active	81	1.51	65.10	0.94	0.93
	Female ADL	Not Active	279				
		Fairly Active	383	−0.65	43.50	0.96	0.96
		Active	282	0.49	54.90	0.94	0.94
		Very Active	56	1.65	66.50	0.96	0.96
	CADL	Not Active	292				
		Fairly Active	382	−0.86	41.40	0.97	0.97
		Active	277	0.60	56.00	0.94	0.94
		Very Active	49	1.9	69.00	0.94	0.94
EAP	Male ADL	Not Active	285				
		Fairly Active	344	−0.64	43.60	0.96	0.96
		Active	256	0.34	53.40	0.92	0.92
		Very Active	115	1.21	62.10	0.91	0.91
	Female ADL	Not Active	285				
		Fairly Active	345	−0.62	43.80	0.96	0.96
		Active	266	0.37	53.70	0.92	0.92
		Very Active	104	1.28	62.80	0.93	0.93
	CADL	Not Active	279				
		Fairly Active	383	−0.65	43.50	0.96	0.96
		Active	282	0.49	54.90	0.94	0.94
		Very Active	56	1.65	66.50	0.96	0.96
MLE	Male ADL	Not Active	285				
		Fairly Active	385	−0.66	43.40	0.96	0.96
		Active	249	0.46	54.60	0.93	0.94
		Very Active	81	1.51	65.10	0.94	0.93
	Female ADL	Not Active	279				
		Fairly Active	383	−0.65	43.50	0.96	0.96
		Active	282	0.49	54.90	0.94	0.94
		Very Active	56	1.65	66.50	0.96	0.96
	CADL	Not Active	289				
		Fairly Active	382	−0.86	41.40	0.97	0.97
		Active	277	0.58	55.80	0.94	0.94
		Very Active	52	1.88	68.80	0.94	0.94

*IRT* Item response theory, *ADL-CAT* activities of daily living-Computerized Adaptive Testing, *CADL* comprehensive activities of daily living-Computerized Adaptive Testing, *MLE* maximum likelihood estimation, *EAP*, expected *a posteriori* estimation, *MAP*, maximum *a posteriori* estimation

*T*-score cutpoints suggested at 45, 55, 65 for ADL scales

### Task 3: Online ADL CAT assessment

**Fig. 3:**
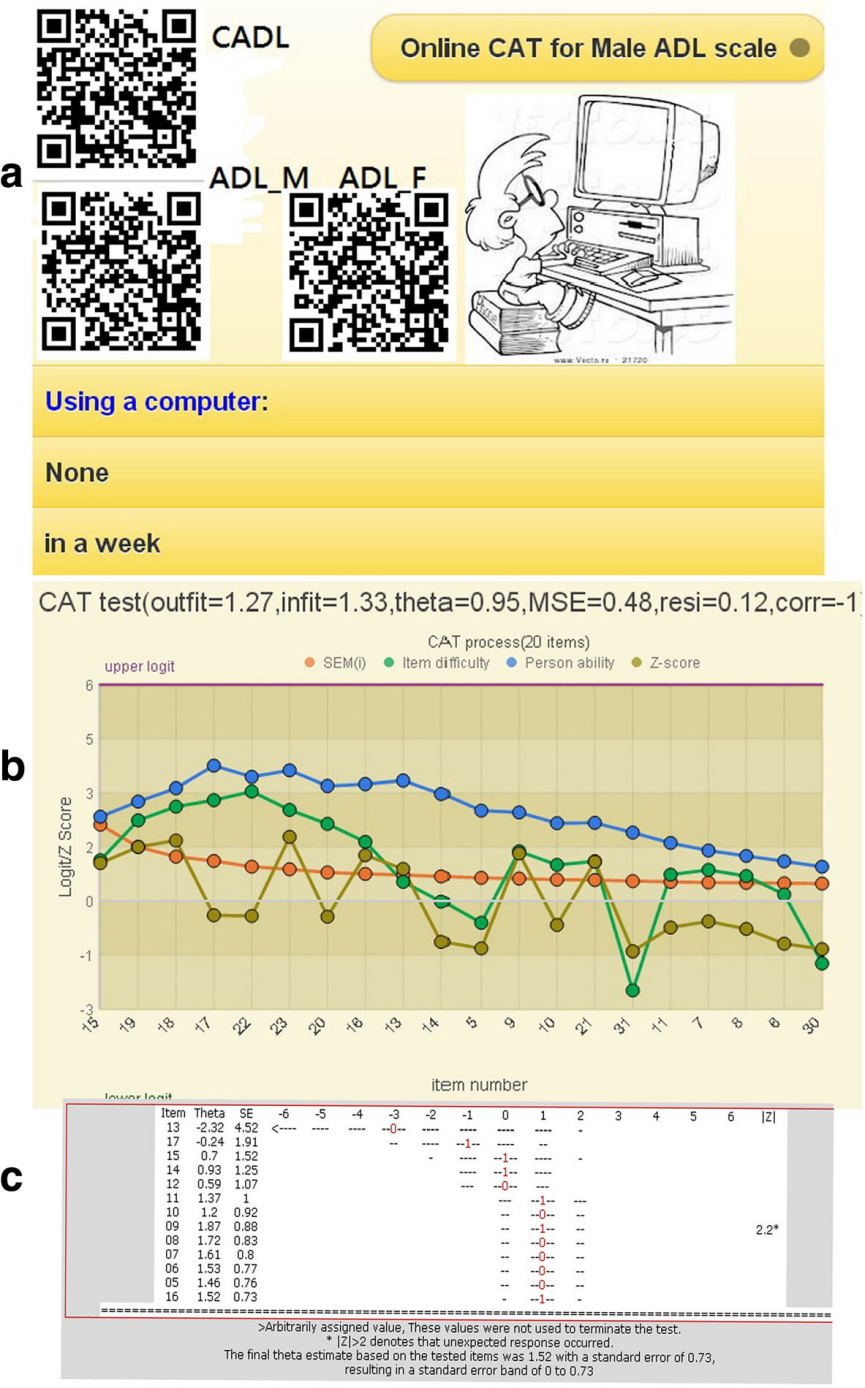
Snapshots (**a**), CAT process (**b**), and the MSE decreased (**c**) when the number of the items increased and an unexpected response with an asterisk (*) when |Z| ≥ 2.0 shown on a smart phone

**Fig. 4:**
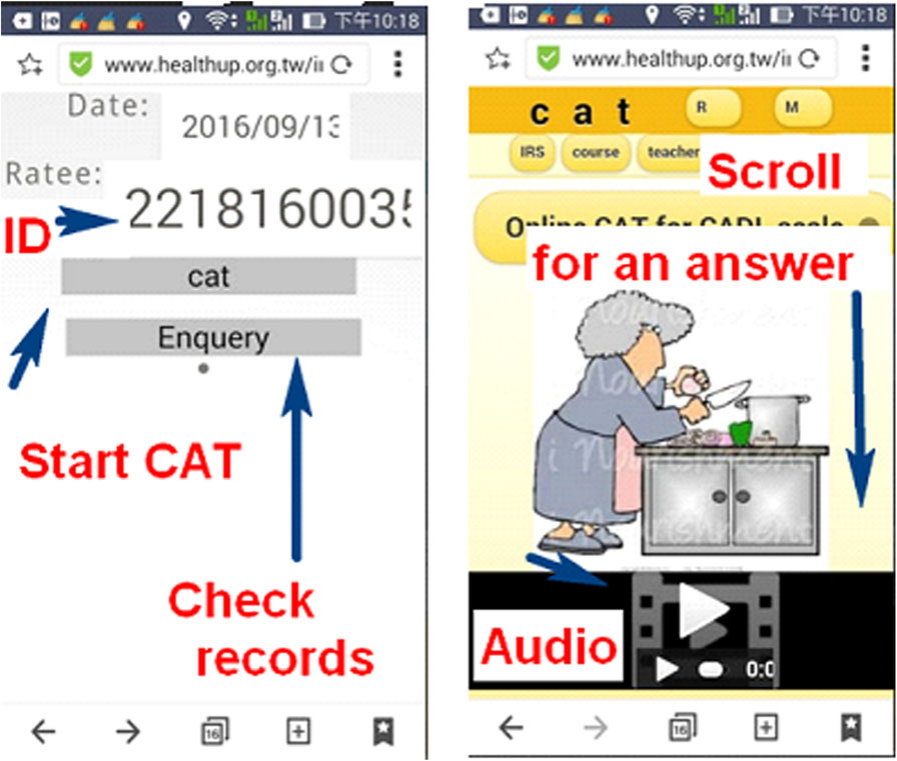
By scanning a QR-code, the first snapshot (left) and the second (right) appear on the smartphone

By scanning a QR-code (Fig. 3a, top left) which encapsulates an appropriate patient ID, the selected ADL CAT appears on the smartphone (left in Fig. 4). We developed a mobile CAT survey procedure to demonstrate practically the newly designed GPCM-type CAT application in action. The CAT processed each ADL item-by-item (Fig. 3b, c). Person fit (i.e., infit and outfit mean-squared error [MNSQ]) statistics showed the respondent behaviors. Person theta is the provisional ability estimated by the CAT module. The MSE in Fig. 3c was generated by this formula:

$$ 1/\surd \left(\varSigma\ \mathrm{information}\ \left(\mathrm{i}\right)\right), $$

where i refers to the finished CAT items responded to by a CAT user [40]. In addition, the resi in Fig. 3b is the average of the last 3 change differences between the pre-and-post estimated abilities on each CAT step. CAT will stop if the resi value is < 0.05. The corr refers to the correlation coefficient between the CAT estimated measures and its step series numbers using the last 5 estimated theta (= person measure) values. The flatter the theta trend, the higher the probability that the person measure is convergent with a final estimation.

## Discussion

### Key findings

Using three CAT estimation methods shows that (i) both CAT and NAT person scores have high correlation coefficients and low different number ratios for the three scales(i.e., all less than 5 %, indicating no difference exists between CAT and NAT), and that the item length is shorter than that of the NAT scores on both the CADL scale and the ADL scale. This implies that CAT is more efficient than NAT without compromising its precision. (ii) The T scores of cutpoints were determined with high specificity and sensitivity (> 0.90), and were suggested at 45, 55, and 65 to separate person ADL function strata with *not active*, *fairly active*, *active*, and *very active*. (iii) An online ADL-CAT graphical representation for smart phones is feasible for classifying ADL strata in patients who have had a stroke.

### What this adds to what was known

Our findings in Task 1 (to compare CAT precision and efficiency) are consistent with the literature [2, 5, 21, 22, 38, 41], and they support the notion that CAT is more efficient than NAT. We confirmed that GPCM-type ADL CAT (i.e., in contrast to CADL-CAT [2, 7], which uses dichotomous Rasch models) similarly requires significantly fewer items for person measures than does NAT, but does not compromise precision of measurement. A clinically useful mobile online assessment APP can be developed for smartphones.

IRT-based CAT is generally different from the traditional pen-and-pencil test for which all items are answered while providing little information to use for analyzing the CAT users’ responses. For instance, outfit MNSQ values of ≥ 2.0 can be a threshold when examining whether patient responses are distorted or abnormal, i.e., whether many responses unexpectedly do not fit the model’s requirements and are deemed highly possibly careless, mistaken, cheating, or awkward [2, 21, 42] (e.g., the outfit MNSQ of 1.27 is shown as controllable, and an unexpected response shows an asterisk (*) on the |Z| column in Fig. 3 if |Z| ≥ 2.0). This is another advantage of IRT over the traditional classic test theory (CTT): it gives more useful information to readers. In addition, any significantly aberrant or cheating behavior on CAT will be detected and found by the CAT module algorithm.

### What it implies and what should be changed

We have provided a way to determine the cutpoints of ADL person strata using a norm-referred method in Task 2. It is because we usually do not know the patient’s true- and false-positive status unless we have applied the so-called gold standard test (i.e., the cutpoint) before the study. Many studies in their Limitations sections caution that their results cannot be generalized to other workplace sites or to other types of patients.

The norm-referred method was thus introduced in this study based on suggestions found in the literature [34–37]. That is to determine the cutpoints of ADL person strata through following stages: Calculating Cronbach’s Alpha of the scale → Computing the number of person strata → Grouping members in each cluster using K-mean statistics → Obtaining means and standard errors for each cluster → Determining cutpoints for each threshold of the sample → Inferring cutpoints to the population.

When we know the means and standard deviations of any two adjacent groups, the cutpoints of person strata can be then determined by using a norm referred method, whereas means are obtained from K-mean cluster analysis, standard deviations are yielded from data of the specific group. The illustration can be seen in worksheet Ch09 in Additional file 1. Through which, the yielded cutpoints can be theoretically generalized to other healthcare sites and other samples when we do not have any idea about the patient’s true- and false-positive disease-specific status.

The *T* scores of cutpoints were then determined. Interested readers are recommended to read Additional file 1 for the detailed calculation and method. Future studies are suggested to use the way to determine cutpoints of malfunction on other clinical functional scales [2–7].

### Strengths of this study

There are two major forms of standardized assessments in clinical settings [43]: (i) a lengthy questionnaire and (ii) a rapid short-form scale [44, 45]. Each has its advantages and drawbacks. However, traditional questionnaires have a large respondent burden because they require patients to answer questions that do not provide any information for the patient estimation [17]. However, we have not seen any online CAT that can be used for smartphones and are suitable for using with MLE, MAP, or EAP on internet.

It is very easy to set up any form (e.g., Rasch partial credit model [PCM] [23] or generalized partial credit model [GPCM] [24]) of online CAT assessment if the designer uploads relevant parameters into the database (e.g., definitions about the type of IRT model; threshold difficulties; the number of questions in the item bank, test, or questionnaire, whether to show plots; etc.). CAT users may expand the item pool or use them in other kinds of scales. It must be said that (i) item overall (i.e., on average) and step (threshold) difficulties of the questionnaire must be calibrated in advance using an IRT model, (ii) pictures and the corresponding audio files used for the subject or response categories for each question should be well-prepared with a web link that can be shown simultaneously with the item appearing in the animation module of CAT, and (iii) the mobile online CAT can be used for many kinds of ITR-based models. The correct parameters corresponding to the exact fields of the database need to be uploaded.

As with all forms of web-based technology, advances in mobile health (mHealth) and health communication technology are rapid [45]. Mobile online CAT is promising and worth promoting the patients' health literacy [46–49]. Interested readers are recommended to see Additional file 4 for the data layout of the online CAT format.

### Limitations and future studies

Our study has some limitations. First, although we, like Hsueh et al. [5], believe that all patients’ true scores follow a normal distribution, there is no evidence to support our assumption in clinical practice, which might influence the determination of cutpoints for the scales. It means that more than one statum is required if data are not normal distributed [37]. We recommend additional studies using other kinds of sample distributions to see whether different cutpoints are arrived at.

Second, although the scale’s Cronbach’s α coefficients were 0.94 for CADL [7] and 0.93 for ADL [5], we conservatively and consistently determined that all the scales’ person strata were four instead of five when Cronbach’s α for CADL reached 0.94 [33] because it is convenient and easy to remember the ADL cutpoints at the *T* scores of 45, 55, and 65.

Third, the study was based on a previously published paper [5]. All of the data were sampled from those released parameters. If any one set (either item or person parameters) were incorrect, the randomized response pattern would be different from the real world. That is, parameters from outpatients living in the community will be different from those of inpatients in a hospital, and those from patients in the chronic stage of stroke will be different from those with other diseases. Usually, the BADL items compared with IADL items are more appropriate for inpatients. The generalizing these ADL-CAT findings might be somewhat limited because of the sample consisted only of inpatients. Additional studies are needed to reexamine whether the psychometric properties of the ADL-CAT suitable only for inpatients, only for patients living in long-term care facilities, or for both.

Fourth, the original ADL-CAT paper assessed gender as a differential item functioning (DIF) factor [50], and proposed two kinds of ADL for males and females, which were used in this study. Thus, the item parameters might be affected by DIF detection for other groups (e.g., living situation).

## Conclusions

We found that ADL-CAT is efficient, reliable, and valid. The online ADL-CAT module used for smartphones is promising for assessing the full spectrum of ADL functions in outpatients with stroke. The visualized presentation of the ADL-CAT module shows that it is feasible for helping both physicians and patients in clinical settings.

## Additional files

Additional file 1: The algorithm for determining Cutpoints and simulating data using MS Excel. (XLS 2362 kb)
Additional file 2: A CAT module in MS Excel. (XLS 6542 kb)
Additional file 3: Comprehensive overview of Rasch models and the CAT process. (PDF 405 kb)
Additional file 4: The file layouts of data used for gathering feedback from patients using online CAT. (XLS 58 kb)

## Abbreviations

ADL: activities of daily living
APP: application
BI: Barthel Index
CADL: comprehensive ADL
CAT: computer adaptive testing
CTT: classic test theory
DC: dimension coefficients
DIF: differential item functioning
EAP: expected a posteriori
FAI: Frenchay Activities Index
GPCM: generalized partial credit model
IRT: Item response theory
MAP: maximum a posteriori
MLE: maximum likelihood estimation
MNSQ: mean-square
MSE: mean-squared error
NAT: Non-Adaptive Testing
PCM: partial credit model
ROC: receiver operating characteristic
SD: standard deviation
SE: standard error
SEM: standard error measurement
VBA: Visual Basic for Applications
